# Pharmacological blockade of the fatty acid amide hydrolase (FAAH) alters neural proliferation, apoptosis and gliosis in the rat hippocampus, hypothalamus and striatum in a negative energy context

**DOI:** 10.3389/fncel.2015.00098

**Published:** 2015-03-27

**Authors:** Patricia Rivera, Laura Bindila, Antoni Pastor, Margarita Pérez-Martín, Francisco J. Pavón, Antonia Serrano, Rafael de la Torre, Beat Lutz, Fernando Rodríguez de Fonseca, Juan Suárez

**Affiliations:** ^1^UGC Salud Mental, Instituto de Investigación Biomédica (IBIMA), Universidad de Málaga-Hospital Universitario Regional de MálagaMálaga, Spain; ^2^CIBER OBN, Instituto de Salud Carlos IIIMadrid, Spain; ^3^Institute of Physiological Chemistry, University Medical Center of the Johannes Gutenberg-University of MainzMainz, Germany; ^4^Institut Hospital del Mar d'Investigacions MediquesBarcelona, Spain; ^5^Facultat de Medicina, Universitat Autonoma de BarcelonaBarcelona, Spain; ^6^Departamento de Biología Celular, Genética y Fisiología, Instituto de Investigación Biomédica (IBIMA), Universidad de MálagaMálaga, Spain; ^7^Facultat de Ciencies de la Salut i de la Vida, Universitat Pompeu Fabra (CEXS-UPF)Barcelona, Spain

**Keywords:** URB597, FAAH, cannabinoids, energy metabolism, neurogenesis, gliosis

## Abstract

Endocannabinoids participate in the control of neurogenesis, neural cell death and gliosis. The pharmacological effect of the fatty acid amide hydrolase (FAAH) inhibitor URB597, which limits the endocannabinoid degradation, was investigated in the present study. Cell proliferation (phospho-H3^+^ or BrdU^+^ cells) of the main adult neurogenic zones as well as apoptosis (cleaved caspase-3^+^), astroglia (GFAP^+^), and microglia (Iba1^+^ cells) were analyzed in the hippocampus, hypothalamus and striatum of rats intraperitoneally treated with URB597 (0.3 mg/kg/day) at one dose/4-days resting or 5 doses (1 dose/day). Repeated URB597 treatment increased the plasma levels of the N-acylethanolamines oleoylethanolamide, palmitoylethanolamide and arachidonoylethanolamine, reduced the plasma levels of glucose, triglycerides and cholesterol, and induced a transitory body weight decrease. The hippocampi of repeated URB597-treated rats showed a reduced number of phospho-H3^+^ and BrdU^+^ subgranular cells as well as GFAP^+^, Iba1^+^ and cleaved caspase-3^+^ cells, which was accompanied with decreased hippocampal expression of the cannabinoid CB1 receptor gene *Cnr1* and *Faah*. In the hypothalami of these rats, the number of phospho-H3^+^, GFAP^+^ and 3-weeks-old BrdU^+^ cells was specifically decreased. The reduced striatal expression of CB1 receptor in repeated URB597-treated rats was only associated with a reduced apoptosis. In contrast, the striatum of acute URB597-treated rats showed an increased number of subventricular proliferative, astroglial and apoptotic cells, which was accompanied with increased *Faah* expression. Main results indicated that FAAH inhibitor URB597 decreased neural proliferation, glia and apoptosis in a brain region-dependent manner, which were coupled to local changes in *Faah* and/or *Cnr1* expression and a negative energy context.

## Introduction

The endocannabinoids participate in a variety of biological activities in the central nervous system, including synaptic communication, neurogenesis (proliferation, differentiation and survival), neuroinflamation and pain perception as a consequence of a neuroprotective response on demand after damage (Stella, 2010; Katona and Freund, 2012; Galve-Roperh et al., 2013; Bandiera et al., 2014). The *N*-acylethanolamines (NAEs), such as arachidonoylethanolamine (AEA), oleoylethanolamine (OEA) and palmitoylethanolamine (PEA), likely belong to independent signaling pathways, including synthesis (NAPE-PLD, PLC, PTPN22, Abh4), inactivation (FAAH, NAAA, COX-2) and cannabinoid (CB1, CB2) and non-cannabinoid (PPARs, GPR55, TRPV1) receptors (Cravatt and Lichtman, 2002; Liu et al., 2006; Mackie and Stella, 2006; Simon and Cravatt, 2006; Yates and Barker, 2009; Ueda et al., 2010). The effectiveness of these endocannabinoids in promoting a specific biological process changes depending on the affinity to their receptors (Pertwee, 2008). Thus, EC_50_ values of AEA are 18, 31, and 27 nM at GPR55, CB1, and CB2 respectively (Devane et al., 1992; Lambert et al., 1999; Ryberg et al., 2007), EC_50_ values of OEA are 0.12, 0.44, >30 and >30 μM at PPARα, GPR55, CB1 and CB2 respectively (Rodríguez de Fonseca et al., 2001), and EC_50_ values of PEA are 0.004, 3, 19.8, and >30 μM at GPR55, PPARα, CB1 and CB2 respectively (Lambert et al., 2001, 2002). The distinct receptor affinity and activation by NAEs signaling can lead to a variety of responses including opposite biological effects. For instance, AEA enhances food intake and energy storage (Jamshidi and Taylor, 2001; Kirkham et al., 2002; Aronne and Thornton-Jones, 2007; Cota, 2008). The activation of CB1 receptors in the paraventricular hypothalamic nucleus and nucleus accumbens increases appetite and eating motivation, respectively (Jamshidi and Taylor, 2001; Kirkham et al., 2002). On the contrary, OEA binds to PPARα to reduce food intake and promote lipolysis (Fu et al., 2003). OEA produces anorexic effects by activating satiety signals forwarded from the vagal afferent neurons to the paraventricular hypothalamic nucleus (Lo Verme et al., 2005a). Contrary to the previous hypothesis, NAEs are able to produce similar biological effects through independent signaling pathways. For instance, both AEA and PEA, targeting to CB1/CB2/TRPV1 and PPARα respectively, can block neuroinflammation by regulating glia migration and survival (Walter et al., 2003; Lo Verme et al., 2005b; Stella, 2010; Stock et al., 2012).

The hydrolyzing NAEs enzyme fatty acid amide hydrolase (FAAH) is expressed in the brain with particularly high levels in the hippocampus and low levels in the hypothalamus (Kano et al., 2009). Moreover, FAAH has different catalytic properties depending on substrate specificity, cell type and physiological conditions (Sun et al., 2005). Despite OEA and PEA are 10 times more abundant than AEA in the brain (Cadas et al., 1997), FAAH preferentially hydrolyzes AEA over OEA and PEA (Ueda et al., 2000). The FAAH inhibitor URB597 (KDS-4103) selectively blocks AEA hydrolysis in neurons without having any inhibitory effect on AEA transport (Kathuria et al., 2003; Piomelli et al., 2006). In rats, at an intraperitoneal dose of 0.3 mg/kg, URB597 is rapid in onset (<15 min), persistent (>12 h) and accompanied by elevations of NAEs in the brain and peripheral tissues (Fegley et al., 2005). Regarding behavior activity, URB597 evoked anxiolytic, antidepressant and antinociceptive-like responses (Clapper et al., 2009; Gaetani et al., 2009; Lomazzo et al., 2015). Through URB597 at a dose of 0.3 mg/kg, it maximally blocks FAAH activity and increases brain levels of AEA, but does not reproduce the spectrum of pharmacological responses that characterize AEA such as catalepsy, hypothermia or hyperphagia, three typical signs of CB1 activation (Kathuria et al., 2003). Furthermore, URB597 was found to promote CB2 antagonist SR144528-sensitive anti-inflammatory effects (Holt et al., 2005), for instance, by decreasing microglial activation in the brain (Murphy et al., 2012; Slusar et al., 2013).

Both CB1 and CB2 receptors can modulate neurogenesis and/or gliogenesis (Aguado et al., 2006; Molina-Holgado et al., 2007; Goncalves et al., 2008; Rivera et al., 2011). CB1 and CB2 activation and FAAH inhibition increased neural proliferation, an action that can be abrogated in CB1-deficient mouse neurospheres and confirmed in the brains of FAAH-deficient mouse embryos (Aguado et al., 2005; Goncalves et al., 2008). Moreover, CB1 activation does not only protect neurons from brain injury (Mechoulam et al., 2002; Parmentier-Batteur et al., 2002; Marsicano et al., 2003), but also protects astrocytes from apoptosis, promotes astroglial proliferation and differentiation, and prevents oligodendrocyte cell death *in vitro* (Aguado et al., 2006; Fernández-Ruiz et al., 2007; Gomez et al., 2011). Interestingly, these effects can be partially reversed by TRPV1 antagonist (Cohen-Yeshurun et al., 2013).

Taken together, the evidence suggests that brain processes such as neurogenesis and neuroprotection can be regulated depending on the endocannabinoid levels and the activation of their local targets. We hypothesized that the FAAH inhibitor URB597 can influence neural proliferation, cell survival and inflammation as a consequence of alterations in the endocannabinoid tone. To this end, we analyzed the cell proliferation and survival in the principal neurogenic zones of the adult rat brain, including the subgranular zone (SGZ) of the dentate gyrus and the subventricular zone (SVZ) of the lateral ventricles, as well as the astroglial, microglial and apoptotic cells in the hippocampus, hypothalamus and striatum of lean rats after the administration of one dose/4-days resting or 5 doses (1 dose/day) of URB597 at an effective dose of 0.3 mg/kg/day. Results were interpreted regarding the energy balance, the plasma levels of OEA, PEA and AEA, and the hippocampal, hypothalamic and striatal expression of FAAH and CB1 receptor.

## Materials and methods

### Ethics statement

The protocols for animal care and use were approved by the Ethics and Research Committee at the Hospital Universitario Regional de Málaga and Universidad de Málaga. All experimental animal procedures were carried out in strict accordance with the European Communities directive 86/609/ECC (24 November 1986) and Spanish legislation (BOE 252/34367-91, 2005) regulating animal research. All efforts were made to minimize animal suffering and to reduce the number of animals used.

### Animals

Male Wistar rats (approximately 250 g, 10–12 weeks old; Charles Rivers, Barcelona, Spain) were housed individually in cages maintained in standard conditions (Servicio de Estabulario, Facultad de Medicina, Universidad de Málaga) at 20 ± 2°C room temperature, 40 ± 5% relative humidity and a 12-h light/dark cycle with dawn/dusk effect. Water and standard rodent chow (Prolab RMH 2500, 2.9 kcal/g) were available *ad libitum*, unless otherwise indicated for specific experimental procedures.

### Habituation

The animals were daily weighed, handled for 10 min and habituated to injection procedures (holding and pseudo-injection) during 1 week before the experimentation in order to minimize stress effects. To further habituate the animals to feeding procedures, 84 h before testing with drug, the animals were food deprived for 24 h with *ad libitum* access to water. Then, the rats were maintained under free-feeding period for 48 h. After this time, the animals were definitively food-deprived for 12 h (with free access to water) before the beginning of the food exposure and treatment. Finally, a can with a measured amount of food (usually 50–60 g per day) and a bottle containing 250 ml of fresh water were placed in the cage at time 0. Food pellets and rats were periodically weighed.

### Administrations of URB597

Fatty acid amide hydrolase (FAAH) inhibitor URB597 (cyclohexyl carbamic acid 3′-carbamoyl-biphenyl-3-yl ester; Cayman Chemical, cat. no. 10046, Ann Arbor, MI, USA) was dissolved in a vehicle containing 33% (*v*/*v*) DMSO in sterile 0.9% NaCl solution, just before each experiment. The vehicle or URB597 were intraperitoneally (i.p.) injected in a final volume of 1 ml/kg body weight. The optimal dose at which acute treatment would be more effective was selected for the repeated treatment experiment (see Supplementary Materials and Figure S1). Thus, URB597 was acutely or repeatedly administrated at a dose of 0.3 mg/kg body weight for 5 consecutive days at intervals of 24 h (08:00 a.m.). During the repeated administration of URB597, the cumulative food intake and body weight gain were daily measured for the 5 days of treatment. In a first batch, the animals were sacrificed 2 h after the last injection of vehicle or URB597. Finally, we generated three experimental groups (*n* = 16/group): (1) Vehicle administration for 5 days (vehicle group); (2) One URB597 administration (0.3 mg/kg/day) and repeated vehicle administration for the remaining 4 days (acute URB597 group); (3) Repeated URB597 administration (0.3 mg/kg/day) for 5 days (repeated URB597 group) (Figure S2). In a second batch, the animals were sacrificed 3 weeks after the last treatment day. We generated two experimental groups (*n* = 8/group): (1) Vehicle administration for 5 days (vehicle group); (2) Repeated URB597 administration (0.3 mg/kg/day) for 5 days (repeated URB597 group) (Figure S2).

### BrdU administration

5′;-bromo-2′-deoxyuridine (BrdU, cat. no. B5002, Sigma, St. Louis, MO, USA) was dissolved at 15 mg/mL in sterile 0.9% NaCl solution, and i.p. administrated at a dose of 50 mg/kg body weight twice per day at intervals of 10 h (08:00, 18:00 h), for 4 consecutive days (Cifuentes et al., 2011).

### Sample collection

Previous to sacrifice, all animals were anaesthetized (sodium pentobarbital, 50 mg/kg body weight, i.p.) 2 h or 3 weeks after the last dose of treatment in a room separate from the other experimental animals. Most blood samples (*n* = 12/group) were briefly collected into tubes containing EDTA-2Na (1 mg/ml blood) and centrifuged (1600 g for 10 min, 4°C). Plasma samples were then frozen and stored at −80°C for biochemical, hormonal and liquid chromatography–mass spectrometry analyses. Half of the first batch of animals (*n* = 8/group) were sacrificed by decapitation and their brains were collected, quick frozen and stored at −80°C. These brains were then prepared on dry ice to obtain sections of 1 mm thick by using razor blades and a rat brain slicer matrix. The hippocampus, hypothalamus and striatum were precisely removed from −2.16 to −4.20 and from 2.28 to −0.24 Bregma levels (Paxinos and Watson, 2007) with fine surgical instruments. Brain samples were weighed and stored at −80°C until they were used for RT-qPCR and liquid chromatography–mass spectrometry analyses. Another half of the first batch of animals and the second batch of animales (*n* = 8/group) were transcardially perfused with 4% formaldehyde in 0.1 M phosphate buffer (PB) and the brains were dissected out and kept in the same fixative solution overnight at 4°C. The brains were then cut into 30-μm-thick coronal sections and were divided in eight parallel series by using a vibratome (Leica VT1000S). Sections were stored at 4°C in PB with 0.002% (*w/v*) sodium azide until they were used for immunostaining.

### Immunohistochemistry

The brain sections were divided in eight parallel series. Each parallel series consisted of one coronal section for each 240 μm. Free-floating coronal sections from −2.16 to −4.20 Bregma levels (hippocampus and hypothalamus), from Bregma 2.28 to -0.24 Bregma levels (striatum) and from 7.60 to 6.60 Begma levels (olfactory bulbs) of each parallel series were selected for each immunohistochemistry. Sections were incubated overnight in the following diluted primary antibody at 4°C: rabbit anti-FAAH (1:200, Cayman, cat no. 101600; Gulyas et al., 2004), rabbit anti-CB1 (1:200, Frontier Institute, cat. no. CB1-Rb-Af380-1; Uchigashima et al., 2007), rabbit anti-phospho-histone H3 (Ser10) (2 μg/ml, Upstate, cat. no. 06-570), mouse anti-BrdU (1:2000, Hybridoma Bank, Iowa City, IA, USA; ref. G3G4), rabbit anti-cleaved caspase-3 (1:500, Cell Signaling; cat. no. 9661), mouse anti-glial fibrillaric acidic protein (GFAP) (1:500, Sigma, cat. no. G3893) and rabbit anti-Iba-1 (1:1000, Wako, cat. no. 019-19741) (Cifuentes et al., 2011; Rivera et al., 2011, 2013). The following day the sections were incubated in the respective secondary antibody for 90 min: biotinylated goat anti-mouse IgG (1:500, Sigma; cat. no. B7264) or biotinylated donkey anti-rabbit IgG (1:500, Amersham, Little Chalfont, England; cat. no. RPN 1004). The sections were then incubated in ExtrAvidin peroxidase (Sigma, St. Louis, MO) diluted 1:2000 in darkness at room temperature for 1 h. Finally, immunolabeling was revealed with 0.05% diaminobenzidine (DAB; Sigma), 0.05% nickel ammonium sulfate and 0.03% H_2_O_2_ in PBS. Brain sections from the second batch of animals were only used to check cell survival by BrdU immunohistochemistry.

### Double immunofluorescence

The following antibodies were used: rat anti-BrdU (1:2000; Accurate Chemical and Scientific, Westbury, NY, USA, cat. no. OBT0030G), mouse anti-NeuN (1:500; Millipore, cat. no. MAB377), mouse anti-β3 tubulin (1:5000; Promega, Madison, WI, USA) and mouse anti-GFAP (1:500; Sigma, cat. no. G3893). Sections were incubated overnight at 4°C with a cocktail of the primary antibodies. The rat anti-BrdU was detected with the donkey anti-rat IgG (H+L) labeled with Alexa Fluor® 488 (1:1000; Molecular Probes, Invitrogen, Paisley, UK, cat. no. A21208). The antibodies NeuN, β3-tubulin and GFAP were detected with the donkey anti-mouse IgG (H+L) labeled with Alexa Fluor® 594 (1:1000; Molecular Probes, cat. no. A21203). The sections labeled by double immunofluorescence (BrdU+NeuN, BrdU+β3-tubulin or BrdU+GFAP) were visualized with a confocal laser (spectral) scanning microscope (Leica TCS NT; Leica Microsystems) equipped with a 561 nm DPM laser (argon 30%) and a 40× objective (HCX PL APO CS 40.0x1.25 OIL UV). The emission filter settings were 500–550 nm for PMT2 (green) and 610–700 nm for PMT3 (red). Depending on the level of zoom used in each image, the XY voxel size was from 100 to 77 nm, approximately. Settings of light and brightness/contrast were adjusted by using the Leica LAS AF Lite imaging software.

### Cell counting

Phospho-H3, BrdU, GFAP, Iba-1 and cleaved caspase 3-immunoreactive (-ir) nuclei and cells that came into focus were manually counted from Bregma −2.16 to −4.20 mm at hippocampal and hypothalamic levels, from Bregma 2.28 to −0.24 mm at striatal levels and from Begma 7.60 to 6.60 mm at olfactory bulb levels (Paxinos and Watson, 2007). Representative counting frames were evaluated using a standard optical microscope with the 40× objective (Nikon Instruments Europe B.V., Amstelveen, The Netherlands) coupled to the NIS-Elements Imaging Software 3.00 (Nikon). Focusing in the hippocampus, phospho-H3 and BrdU-ir nucleus counts were performed in the subgranular zone (SGZ) of the dentate gyrus (DG), while survived BrdU-ir nucleus counts and GFAP, Iba-1 and cleaved caspase 3-ir cell counts were carried out in the whole hippocampus (DG, CA3 and CA1 areas). Focusing in the hypothalamus, counting was performed in the paraventricular (PVH), ventromedial (VMH) and arcuate (ARC) nuclei and median eminence. Focusing in the striatum, phospho-H3 and BrdU-ir nucleus counts were performed in the subventricular zone (SVZ) of the lateral ventricles, while survived BrdU-ir nucleus counts and GFAP, Iba-1 and cleaved caspase 3-ir cell counts were carried out in the whole striatum. Survived BrdU-ir nucleus counts were also performed in the olfactory bulbs. Immunostained cells located in the uppermost side that came into focus while moving down through the thickness of the section were counted. Overall, quantification was expressed as the average number of cells per area (mm^2^) for each experimental group.

### Quantification of immunoreactivity

Densitometric analysis of the GFAP, FAAH, and CB1 immunoreactivity was evaluated in the three hippocampal areas (dentate gyrus, CA3 and CA1) and hypothalamus from Bregma −2.16 to −4.20 mm, and in the striatum from Bregma 2.28 to −0.24 mm (Paxinos and Watson, 2007). Digital high-resolution microphotographs of representative areas were taken with a 10× objective under the same conditions of light and brightness/contrast with an Olympus BX41 microscope equipped with an Olympus DP70 digital camera. Quantification was determined using the analysis software ImageJ 1.38X (NIH, USA). Focusing in CA1 and CA3 areas, we considered the following layers: stratum oriens (SO), stratum pyramidale (SP), stratum radiatum (SR), stratum lucidum (SL) and stratum lacunosum-moleculare (SL-M). For dentate gyrus, we considered the molecular layer (ml), the granular cell layer (gcl) and the polymorphic cell layer (pcl). Focusing in the hypothalamus, densitometry were performed in the paraventricular (PVH), ventromedial (VMH) and arcuate (ARC) nuclei and median eminence.

### RNA isolation and RT-qPCR analysis

We performed real-time PCR (TaqMan, Applied Biosystem, Carlbad, CA, USA) as described previously (Crespillo et al., 2011; Decara et al., 2012) using specific sets of primer probes (*Faah*: Rn00577086_m1, Amplicon length: 63; *Cnr1*: Rn02758689_s1, Amplicon length: 92; *Actb*: Rn00667869_m1, Amplicon length: 91; Life Technologies). Briefly, brain regions were homogenized on ice and RNA was extracted following Trizol® method according to the manufacture's instruction (Gibco BRL Life Technologies, Baltimore, MD, USA). RNA samples were isolated with RNAeasy minelute cleanup-kit including digestion with DNase I column (Qiagen, Hilden, Germany). After reverse transcript reaction from 1 μg of mRNA, quantitative real-time reverse transcription polymerase chain reaction (qPCR) was performed in a CFX96TM Real-Time PCR Detection System (Bio-Rad, Hercules, CA, USA) and the FAM dye label format for the TaqMan® Gene Expression Assays (Applied Biosystem). Melting curve analysis was performed to ensure that only a single product was amplified. After analyzing several control genes, values obtained from the brain samples were normalized in relation to β-actin (*Actb*) levels.

### Endocannabinoid quantification

The determination of AEA, PEA, OEA, and 2-arachidonoyl glycerol (2-AG) was done as previously described (Pastor et al., 2014). Briefly, aliquots of 175 μL of plasma were spiked with AEA-d4, PEA-d4, OEA-d4, and 2-AG-d5 (Cayman Chemical, Ann Harbor, MI), diluted up to 1 mL with 0.1M ammonium acetate pH 4.0 (Merck, Darmstadt, Germany), extracted with tert-butyl-methyl-ether (Merck) and analyzed by LC/MS-MS on a triple quadrupole mass spectrometer (Agilent 6410, Wilmington, DE) that operated with an electrospray ionization source (ESI) on the positive mode. Chromatographic separation was done with a Waters C18-CSH column (3.1 × 100 mm × 1.8 μm particle size), detection was done by selected reaction monitoring (SRM) and quantification was done by isotope dilution. 2-MGs were reported as the sum of isomer 1 and isomer 2 due to the instability of isomer 2 to isomerization.

### Biochemical and hormonal analysis

Glucose, triglycerides, total cholesterol, high-density lipoprotein (HDL)-cholesterol, creatinine, urea, uric acid and the hepatic enzymes aspartate transaminase (AST) and alanine transaminase (ALT) were analyzed using commercial kits according to the manufacturer's instructions in a Hitachi 737 Automatic Analyzer (Hitachi Ltd., Tokyo, Japan). Plasma levels of IGF-1, insulin, leptin and adiponectin were determined with three different enzyme-linked immunosorbent assay (ELISA) kits from BioVendor (Modrice, Czech Republic), Mercodia AB (Uppsala, Sweden) and B-Bridge International, Inc. (Mountain View, CA, USA), respectively. To perform the ELISA protocols in rat samples, we used 50, 25, 100, 100 μl of plasma to determine the concentrations of IGF-1, insulin, leptin, adiponectin, respectively. In all cases, a calibration curve and internal controls were included in each assay.

### Statistical analysis

Statistical analysis of the results was performed using the computer program GraphPad Prism version 5.04 (GraphPad Software Inc., San Diego, CA, USA). Data were represented as the mean ± s.e.m. for at least six determinations per experimental group. Kolmogorov-Smirnov normality tests indicated that all data followed a Gaussian distribution (*P* > 0.1), so we selected a parametric statistical test. Statistical analysis was performed using one or Two-Way ANOVA and Bonferroni's test or Student's *t*-test when appropriate. *P* < 0.05 was considered to be significant.

## Results

### Metabolic status of the rats treated with URB597

**Figure 1:**
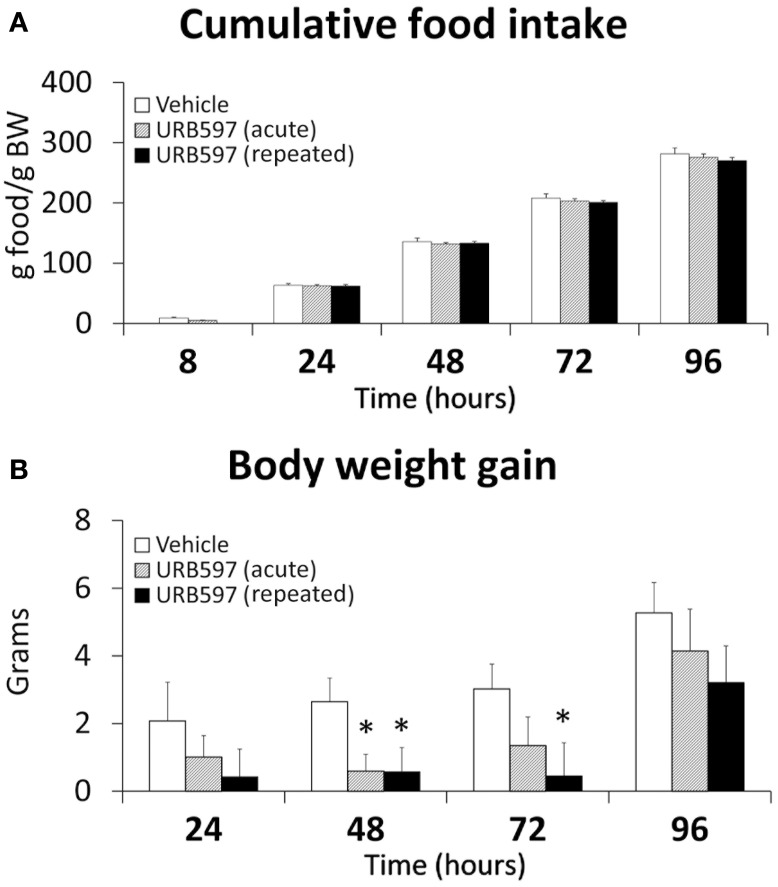
**Effect of acute and repeated treatment of URB595 (0.3 mg/kg) on cumulative food intake (A) and body weight gain (B)**. The histograms represents the mean ± s.e.m. (*n* = 8) per experimental group. ANOVA and Bonferroni's test: ^*^P < 0.05 vs. vehicle group.

We analyzed the effect of URB597 on food intake and body weight gain for 5 days (Figure 1). After Two-Way ANOVA analysis, a treatment effect on body weight gain was observed [*F*_(2, 84)_ = 5.89, *P* = 0.004]. Moreover, the effect of the repeated treatment on body weight gain showed a higher significance [*F*_(1, 56)_ = 10.7, *P* = 0.001] than that of the acute treatment [*F*_(1, 56)_ = 5.82, *P* = 0.019]. As a consequence, repeated URB597-treated rats showed a decrease in their body weight gain up to 48 and 72 h after the beginning of the repeated treatment when they were compared with vehicle-treated rats (^*^P < 0.05) (Figure 1B). Moreover, a transitory decrease in the body weight gain was also observed in the second day after acute treatment of URB597.

### Biochemical and hormonal profile of the rats treated with URB597

To better understand the metabolic context associated to URB597 treatment, we evaluated the biochemical and hormonal profile in the plasma of these rats (Table 1). Biochemical parameters included basal glucose, triglycerides, total cholesterol, high-density lipoprotein (HDL)-cholesterol, creatinine, urea, uric acid and aspartate transaminase (AST)/alanine transaminase (ALT) ratio. Metabolic hormones included insulin, leptin, adiponectin and IGF-1. The plasma of rats acutely and repeatedly treated with URB597 showed lower levels of basal glucose [*F*_(2, 15)_ = 5.95, ^*^P < 0.05], triglycerides [*F*_(2, 15)_ = 6.69, ^*^P < 0.05], total cholesterol [*F*_(2, 15)_ = 6.29, ^*^P < 0.05], HDL-cholesterol [*F*_(2, 15)_ = 5.21, ^**^P < 0.01 and ^*^P < 0.05, respectively] and urea [*F*_(2, 15)_ = 5.22, ^*^P < 0.05] (Table 1). The AST/ALT ratio, used as diagnostic marker for liver damage, was lower in the plasma of repeated URB597-treated rats than that of vehicle or acute URB597-treated rats [*F*_(2, 15)_ = 7.13, ^**/##^*P* < 0.01]. URB597 didn't induce any change in the plasma levels of uric acid. Regarding insulin, we observed a decrease in the plasma levels of the rats repeatedly treated with URB597 [*F*_(2, 15)_ = 5.03, ^**^P < 0.01]. The decrease of both basal glucose and insulin levels in plasma resulted in reduced values of the Homeostatic Model Assessment (HOMA)-IR [*F*_(2, 15)_ = 8.44, ^**^P < 0.01], defined as an index for insulin resistance, as well as increased values of HOMA-β [*F*_(2, 15)_ = 7.74, ^**^P < 0.01], defined as an index for beta-cell function, in the rats acutely and repeatedly treated with URB597 (Table 1). In addition, leptin levels were significantly reduced only in the plasma of the repeated URB597-treated rats [*F*_(2, 15)_ = 25.22, ^***^P < 0.001]. No effects were detected in the plasma levels of adiponectin and IGF-1 after URB597 treatment (Table 1).

**Table 1:** **Effect of URB597 on plasma metabolites and hormones^a^**.

	**Vehicle**	**URB597 (acute)**	**URB597 (repeated)**
Glucose (md/dL)	165.8±7.1	131.8±13.8^*^	131.9±9.5^*^
Triglycerides (mg/dL)	153.0±22.2	88.0±4.2^*^	101.6±11.6^*^
Total cholesterol (mg/dL)	87.6±2.5	78.0±2.2^*^	78.7±2.2^*^
HDL-cholesterol (mg/dL)	27.0±0.4	24.6±0.6^**^	24.8±1.1^*^
Creatinine (mg/dL)	0.56±0.01	0.58±0.02	0.56±0.02
Urea (mg/dL)	37.2±0.8	33.1±1.1^*^	33.5±1.09^*^
Uric acid (mg/dL)	0.63±0.03	0.70±0.03	0.66±0.02
AST/ALT ratio	0.35±0.05	0.34±0.05	0.18±0.02^**^^##^
Insulin (ng/mL)	4.62±1.03	2.7±0.79	1.43±0.37^**^
HOMA-IR (a.u.)	41.3±9.2	17.8±4.6^*^	10.1±2.5^**^
HOMA-β (a.u.)	254.7±25.1	485.5±64.7^**^	515.5±68.1^**^
Leptin (pg/mL)	4130| 269	4219±177	2393±147^***^
Adiponectin (ng/mL)	7168±561	8864±551	6763±834
IGF-1 (ng/mL)	1285±37	1270±37	1268±67

a Levels of plasma metabolites and metabolic hormones in rats acutely (one dose/4-days resting) and repeatedly (5 doses) treated with URB597 (0.3 mg/kg). Values represent the mean ± s.e.m. (n = 6). One-way ANOVA:

* *P < 0.05*,

** *P < 0.01*,

*** P < 0.001 vs. rats treated with vehicle;

## *P < 0.05 vs. rats acutely treated with URB597*.

### Effect of URB597 on cell proliferation in the subgranular zone of the dentate gyrus, the hypothalamus and the subventricular zone of the lateral ventricles

**Figure 2:**
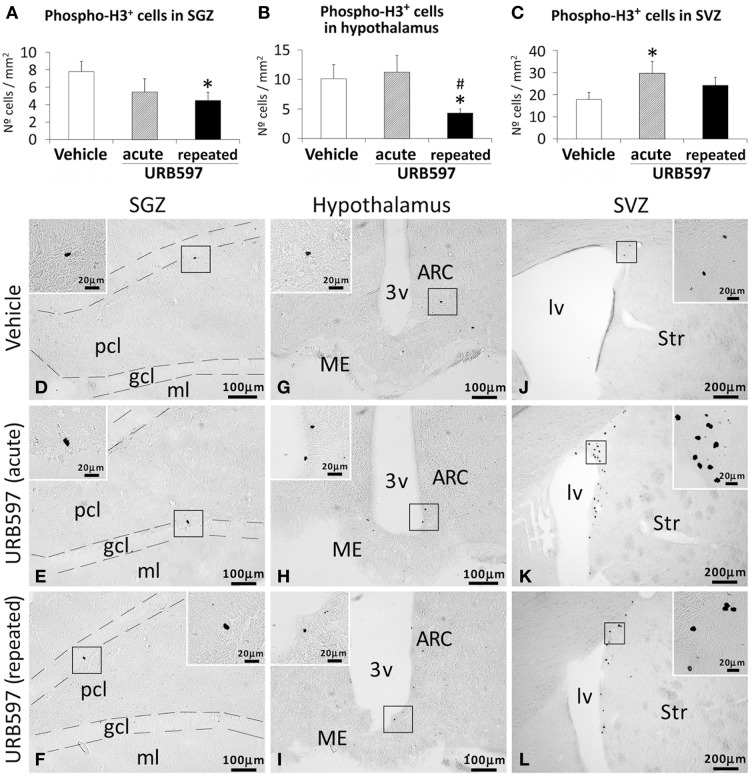
**Effects of acute and repeated treatment of URB595 (0.3 mg/kg) on mitosis in the SGZ, hypothalamus and SVZ by phospho-histone H3 immunohistochemistry**. **(A–C)** The number of positive cells is significantly lower in the SGZ and hypothalamus of repeated URB597-treated rats, and significantly higher in the SVZ of acute URB597-treated rats. **(D–L)** Representative microphotographs showing low and high (insets) magnification views of the typical mitotic cells expressing phospho-H3. The histograms represents the mean ± s.e.m. per area (mm^2^) (*n* = 6–8) of the number of phospho-H3^+^ cells per experimental group. ANOVA and Bonferroni's test: ^*^P < 0.05 vs. vehicle group, ^#^*P* < 0.05 vs. acute URB597 group. Scale bars are included in each image.

**Figure 3:**
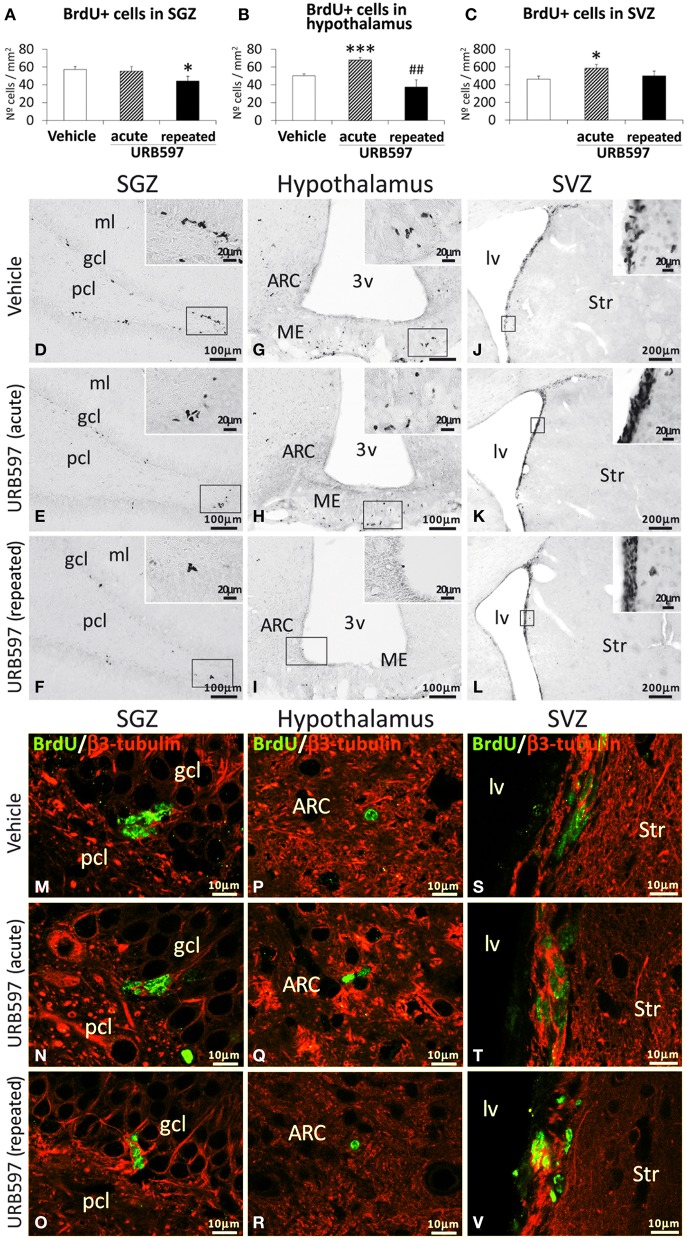
**Effects of acute and repeated treatment of URB595 (0.3 mg/kg) on cell proliferation in the SGZ, hypothalamus and SVZ by BrdU immunohistochemistry**. **(A–C)** The number of positive cells is significantly lower in the SGZ of repeated URB597-treated rats, and significantly higher in the hypothalamus and SVZ of acute URB597-treated rats. **(D–L)** Representative microphotographs showing low and high (insets) magnification views of the typical clustering of newborn cells containing the BrdU labeling. **(M–V)** Newborn cells in the SGZ, hypothalamus and SVZ showing the co-localization of BrdU (green) and the neuron-specific β3-tubulin (red) by confocal immunofluorescence. The histograms represents the mean ± s.e.m. per area (mm^2^) (*n* = 6–8) of the number of BrdU^+^ cells per experimental group. ANOVA and Bonferroni's test: ^*^P < 0.05, ^***^P < 0.001 vs. vehicle group, ^##^*P* < 0.01 vs. acute URB597 group. Scale bars are included in each image.

To investigate the impact of URB597 (0.3 mg/kg) on cell proliferation in relevant neurogenic zones of the adult brain, we evaluated the number of newborn cells in the SGZ, hypothalamus and SVZ by the analysis of the mitosis-related protein phospho-histone H3 (Figure 2) and after the administration of BrdU (50 mg/kg) for 4 days (Figure 3). The number of phospho-H3 and BrdU-ir cells was differentially detected depending on the dose and the neurogenic zone analyzed. The number of phospho-H3-ir cells was lower in the SGZ and hypothalamus after repeated URB597 administration [*F*_(2, 19)_ > 7.13, ^*^P < 0.05] (Figures 2A,B,D–I), but higher in the SVZ after acute URB597 administration [*F*_(2, 21)_ = 4.24, ^*^P < 0.05] (Figures 2C,J–L) compared with the vehicle group. Equally, the number of BrdU-ir cells was lower in the SGZ after repeated URB597 [*F*_(2, 15)_ = 5.1, ^*^P < 0.05] (Figures 3A,D–F) and higher in the SVZ after acute URB597 [*F*_(2, 15)_ = 4.43, ^*^P < 0.05] (Figures 3C,J–L). Contrary to phospho-H3, a significant increase in the number of BrdU-ir cells was detected in the hypothalami of acute URB597-treated rats [*F*_(2, 15)_ = 15.21, ^***^P < 0.001] (Figures 3B,G–I). Moreover, no change in the number of BrdU-ir cells was observed in the hypothalami of repeated URB597-treated rats compared with the vehicle group, but it was significantly reduced compared with the acute URB597-treated rats [*F*_(2,15)_ = 15.21, ^##^*P* < 0.01].

To determine the putative neuronal linage of these BrdU-ir cells, we also evaluated the presence of their co-expression with the neuron-specific β3-tubulin. As is shown in the representative images of Figures 3M–V, we can observe some co-localization of BrdU and neuron-specific β3-tubulin in a number of cells in the SGZ (Figures 3M–O), hypothalamus (Figures 3P–R) and SVZ (Figures 3S–V) of the three experimental groups. These data indicate a neuronal linage of at least part of the newborn cells after 5 days of BrdU treatment.

### Effect of URB597 on cell survival in the hippocampus, hypothalamus, striatum, and olfactory bulbs

**Figure 4:**
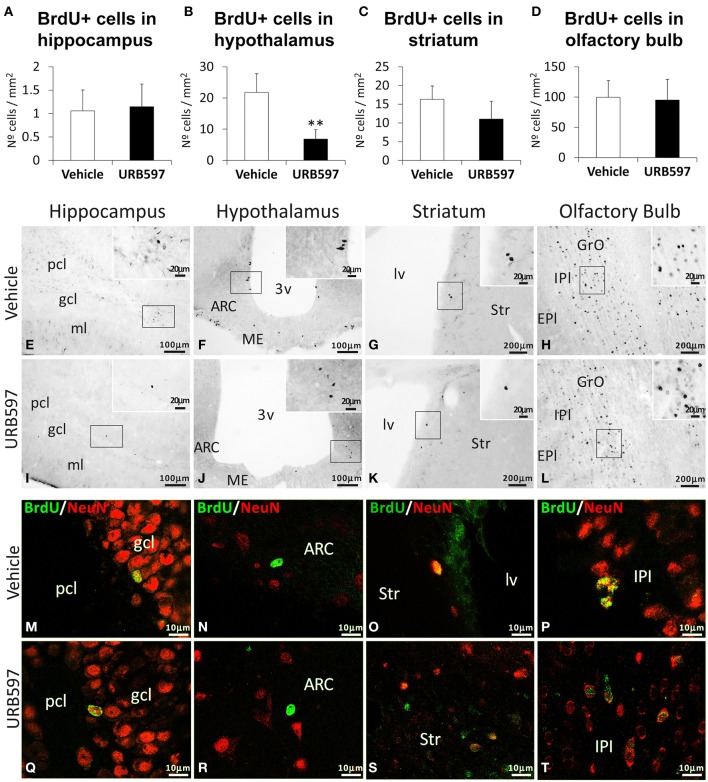
**Effects of repeated treatment of URB595 (0.3 mg/kg) on cell survival in the hippocampus, hypothalamus, striatum and olfactory bulbs by BrdU immunohistochemistry**. **(A–D)** The number of positive cells is significantly lower in the hypothalamus of repeated URB597-treated rats sacrificed 3 weeks after BrdU administration. **(E–L)** Representative microphotographs showing low and high (insets) magnification views of the typical clustering of BrdU-ir cells. **(M–T)** Cells in the hippocampus, hypothalamus, striatum and olfactory bulbs showing the co-localization of BrdU (green) and the biomarker for mature neurons NeuN (red) by confocal immunofluorescence. The histograms represents the mean ± s.e.m. per area (mm^2^) (*n* = 6–8) of the number of BrdU^+^ cells per experimental group. ANOVA and Bonferroni's test: ^**^P < 0.01 vs. vehicle group. Scale bars are included in each image.

To investigate the impact of URB597 on cell survival, we evaluated the number of BrdU-ir cells in the hippocampus, hypothalamus, striatum and olfactory bulbs of rats sacrificed 3 weeks after administering BrdU (Figure 4). The hippocampus, striatum and olfactory bulbs of rats repeatedly treated with URB597 did not show any difference in the number of BrdU-ir cells compared with those of vehicle-treated rats (Figures 4A,C,D). However, we observed a significant decrease in the number of BrdU-ir cells specifically in the hypothalami of URB597-treated rats [*F*_(1, 6)_ = 11.43, ^**^P < 0.01] (Figure 4B).

To determine whether these BrdU-ir cells are neurons, we analyzed their co-expression with the biomarker for mature neurons NeuN (Figures 4M–T). We observed co-localization of BrdU and NeuN in a number of cells in the hippocampus (mainly in the granular cell layer) (Figures 4M,Q), striatum (Figures 4O,S) and olfactory bulb (mainly in the internal plexiform and granule cell layers) (Figures 4P,T) of the two experimental groups. In contrast, we could not detect BrdU/NeuN^+^ cells in the hypothalamic areas analyzed (Figures 4N,R).

### Effect of URB597 on astroglia in the hippocampus, hypothalamus and striatum

**Figure 5:**
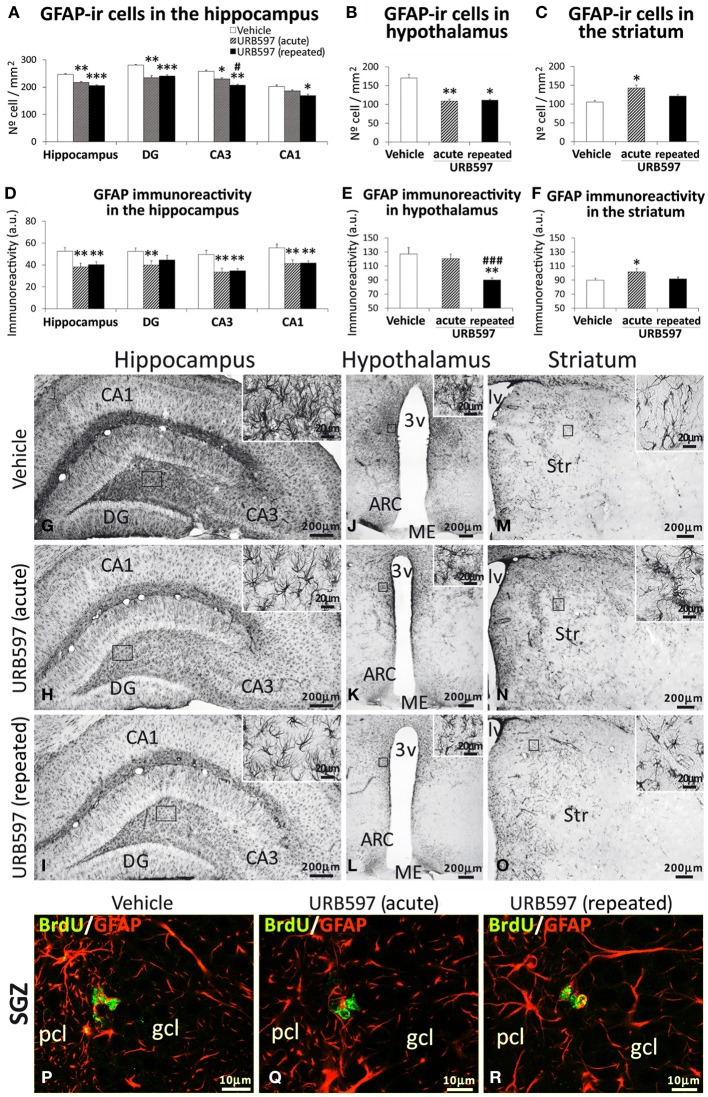
**Effects of acute and repeated treatment of URB595 (0.3 mg/kg) on astroglia in the SGZ, hypothalamus and SVZ by GFAP immunohistochemistry**. **(A–F)** The number of positive cells and the intensity of GFAP immunoreactivity are significantly lower in the hippocampus and hypothalamus of repeated URB597-treated rats, and significantly higher in the striatum of acute URB597-treated rats. **(G–O)** Representative microphotographs showing low and high (insets) magnification views of cells expressing GFAP. **(M–V)** Newborn cells in the SGZ showing the co-localization of BrdU (green) and the astrocyte-specific GFAP (red) by confocal immunofluorescence. The histograms represents the mean ± s.e.m. per area (mm^2^) (*n* = 6–8) of the number of GFAP^+^ cells per experimental group. ANOVA and Bonferroni's test: ^*^P < 0.05, ^**^P < 0.01, ^***^P < 0.001 vs. vehicle group, ^#^*P* < 0.05, ^###^*P* < 0.001 vs. acute URB597 group. Scale bars are included in each image.

To investigate the impact of URB597 on astroglia, we evaluated the intensity of GFAP immunoreactivity and the number of astrocytes that express GFAP in the hippocampus, hypothalamus and striatum (Figure 5). The hippocampi and hypothalami of rats acutely or repeatedly treated with URB597 showed an overall decrease in the number of GFAP-ir cells compared with those of vehicle-treated rats [Hippocampus, one-dose: *F*_(2,21)_ = 12.89, ^**^P < 0.01, 5-doses: ^***^P < 0.001; Hypothalamus, one-dose: *F*_(2, 21)_ = 9.17, ^**^P < 0.01, 5-doses: ^*^P < 0.05] (Figures 5A,B). Regarding the hippocampus, the reduction in the number of cells expressing GFAP was significant in the dentate gyrus (^***^P < 0.001), CA3 (^**^P < 0.01) and CA1 (^*^P < 0.05) fields. This decrease in the number of GFAP-ir cells agrees with a lower immunoreactivity of GFAP in both brain regions (Hippocampus, acute and repeated URB597: ^**^P < 0.01; Hypothalamus, repeated URB597: ^**^P < 0.01) (Figures 5D,E). It should be noted that the decrease of GFAP immunoreactivity in the hippocampus and hypothalamus after URB597 treatment can be also attributed as a consequence of a reduction in the density of branched processes that characterize the labeled astrocytes (Figures 5G–L, insets). This effect contrasted with the increase in the number of GFAP-ir cells and GFAP immunoreactivity observed in the striatum of acute URB597-treated rats [*F*_(2, 18)_ = 5.11,^*^P < 0.05] (Figures 5C,F,M–O). However, when the treatment of URB597 was prolonged for 5 days, we could not observe any change in the number of GFAP-ir cells or GFAP immunoreactivity in the striatum.

Focusing on the SGZ of the dentate gyrus, we observed the presence of some BrdU-ir cells that expressed GFAP (Figures 5P–R) in the three experimental groups. These data can suggest a glial linage of part of the newborn cells in the SGZ after 5 days of BrdU treatment. We could not find co-localization of BrdU and GFAP in cells of the hypothalamus and striatum.

### Effect of URB597 on microglia in the hippocampus, hypothalamus and striatum

**Figure 6:**
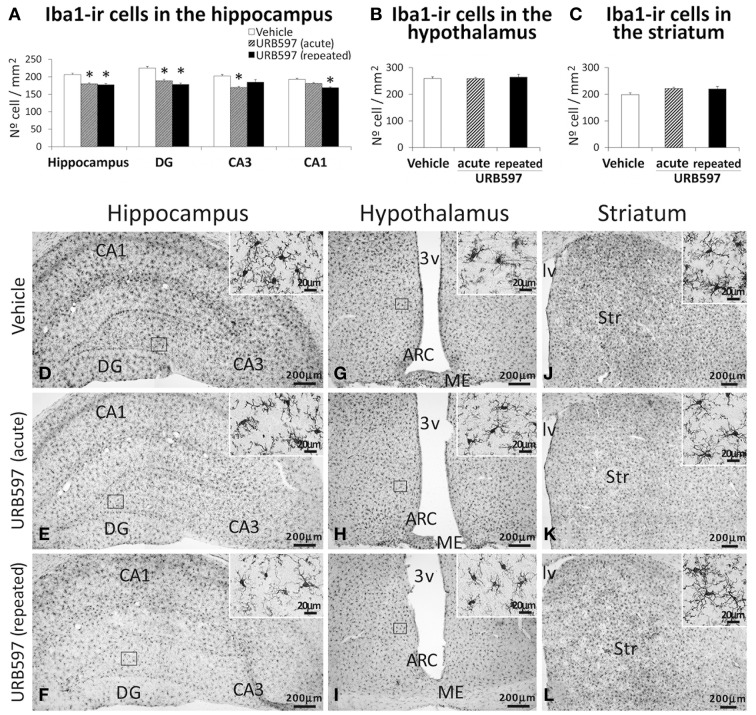
**Effects of acute and repeated treatment of URB595 (0.3 mg/kg) on microglia in the SGZ, hypothalamus and SVZ by Iba-1 immunohistochemistry**. **(A–C)** The number of positive cells are specifically lower in the hippocampus of acute and repeated URB597-treated rats. **(D–L)** Representative microphotographs showing low and high (insets) magnification views of cells expressing Iba-1. The histograms represents the mean ± s.e.m. per area (mm^2^) (*n* = 6–8) of the number of Iba1^+^ cells per experimental group. ANOVA and Bonferroni's test: ^*^P < 0.05 vs. vehicle group. Scale bars are included in each image.

To investigate the impact of URB597 on microglia, we evaluated the number of microglial cells that express Iba1 in the hippocampus, hypothalamus and striatum (Figure 6). The hippocampus of rats acutely and repeatedly treated with URB597 showed an overall decrease in the number of cells expressing Iba1 compared with that of vehicle-treated rats [*F*_(2, 17)_ = 7.12, ^*^P < 0.05] (Figures 6A,D–F). Iba1-ir cells in the hippocampus of repeated URB597-treated rats also showed an apparent reduction in the density of branched processes that characterize the microglial cells (Figures 6D–F, insets). On the contrary, no effect was observed in the hypothalamus and striatum of the acutely and repeatedly treated rats (Figures 6B,C,G–L).

### Effect of URB597 on apoptotic cells in the hippocampus, hypothalamus, and striatum

**Figure 7:**
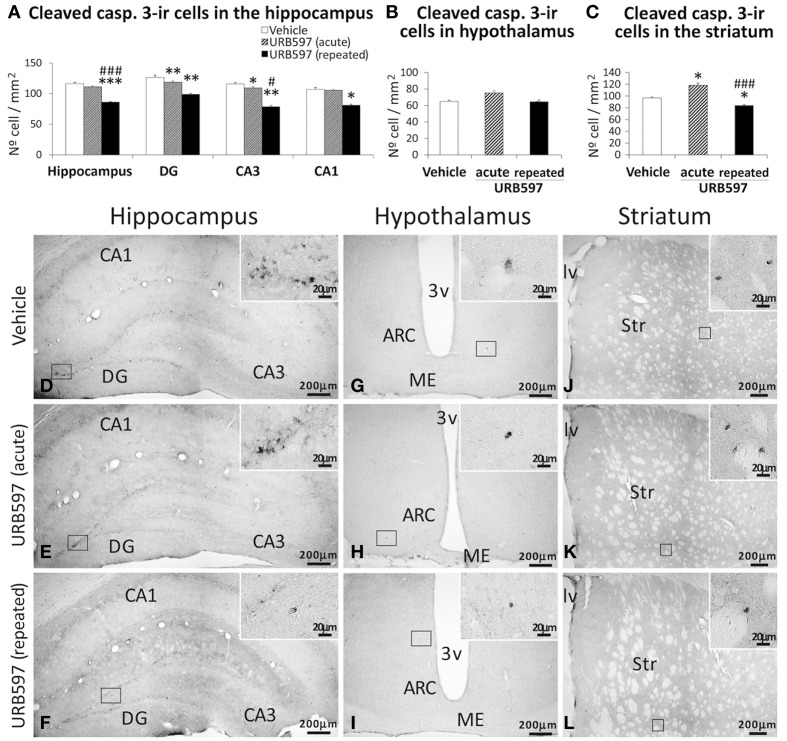
**Effects of acute and repeated treatment of URB595 (0.3 mg/kg) on apoptosis in the SGZ, hypothalamus and SVZ by cleaved caspase-3 immunohistochemistry**. **(A–C)** The number of positive cells are significantly lower in the hippocampus and striatum of repeated URB597-treated rats, and significantly higher in the striatum of acute URB597-treated rats. **(D–L)** Representative microphotographs showing low and high (insets) magnification views of cells expressing cleaved caspase-3. The histograms represents the mean ± s.e.m. per area (mm^2^) (*n* = 6–8) of the number of cleaved casp. 3^+^ cells per experimental group. ANOVA and Bonferroni's test: ^*^P < 0.05, ^**^P < 0.01, ^***^P < 0.001 vs. vehicle group, ^#^*P* < 0.05, ^###^*P* < 0.001 vs. acute URB597 group. Scale bars are included in each image.

Apoptotic cells, as determined by cleaved caspase-3, were assessed in the hippocampus, hypothalamus and striatum of rats acutely and repeatedly treated with URB597 (Figure 7). Differences in the number of cleaved caspase-3-ir cells were detected depending on the dose and brais zone analyzed. The hippocampus of rats treated with repeated URB597 showed an overall decrease in the number of cleaved caspase-3-ir cells compared with that of vehicle-treated rats [*F*_(2, 19)_ = 21.31, ^***^P < 0.001] (Figures 7A,D–F). The decrease in the number of apoptotic cells was significant in the dentate gyrus, CA3 (both at ^**^P < 0.01) and CA1 (^*^P < 0.05), being especially relevant in those rats acutely treated with URB597 (DG: ^**^P < 0.01; CA3: ^*^P < 0.05). No changes in the number of cells expressing cleaved caspase-3 were observed in the hypothalamus (Figures 7B,G–I). Opposed effects were observed in the striatum depending on the dose of URB597 used (Figures 7C,J–L). Thus, as was described above for BrdU and GFAP, we observed an increase in the number of cleaved caspase-3-ir cells in the striatum of acute URB597-treated rats compared with vehicle-treated rats [*F*_(2, 20)_ = 12.17, ^*^P < 0.05]. On the contrary, the striatum of the rats repeatedly treated with URB597 showed a decrease in the number of cells expressing cleaved caspase-3 compared with that of the rats treated with vehicle (^*^P < 0.05) or after 5 days of the acute URB597 (^###^*P* < 0.001) (Figures 7C,J–L).

### Effect of URB597 on the plasma levels of endocannabinoids

**Figure 8:**
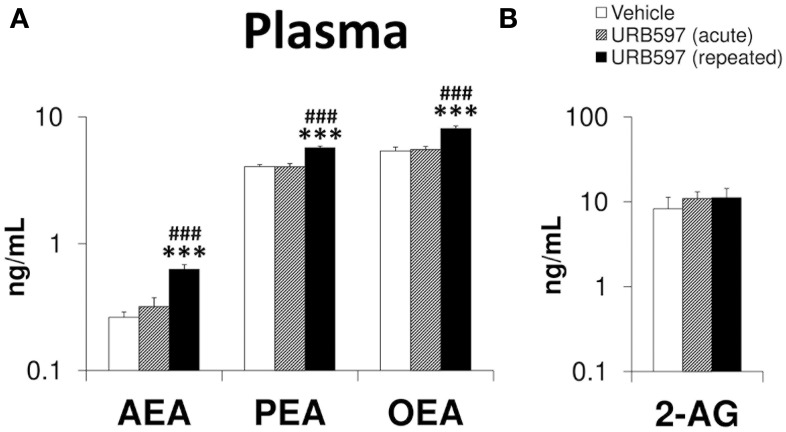
**Effects of acute and repeated treatment of URB595 (0.3 mg/kg) on the plasma levels of AEA, PEA, OEA (A) and 2-AG (B)**. The histograms represents the mean ± s.e.m. (*n* = 12) per experimental group. ANOVA and Bonferroni's test: ^***^P < 0.001 vs. vehicle group, ^###^*P* < 0.001 vs. acute URB597 group.

To determine the endocannabinoid tone after the URB597 treatment, we analyzed the levels of AEA, PEA, OEA, and 2-AG in the plasma (Figure 8). As we expected, increased levels of AEA, PEA, and OEA were exclusively observed in the plasma of repeated URB597-treated rats compared with vehicle [*F*_(2, 33)_ > 17.38, ^***^P < 0.001] or acute URB597 (^###^*P* < 0.001)-treated rats (Figure 8). Neither of the two administrations used for URB597 modified the plasma levels of 2-AG.

### Effect of URB597 on the gene and immunohistochemical expression of FAAH and CB1 receptor in the hippocampus, hypothalamus and striatum

**Figure 9:**
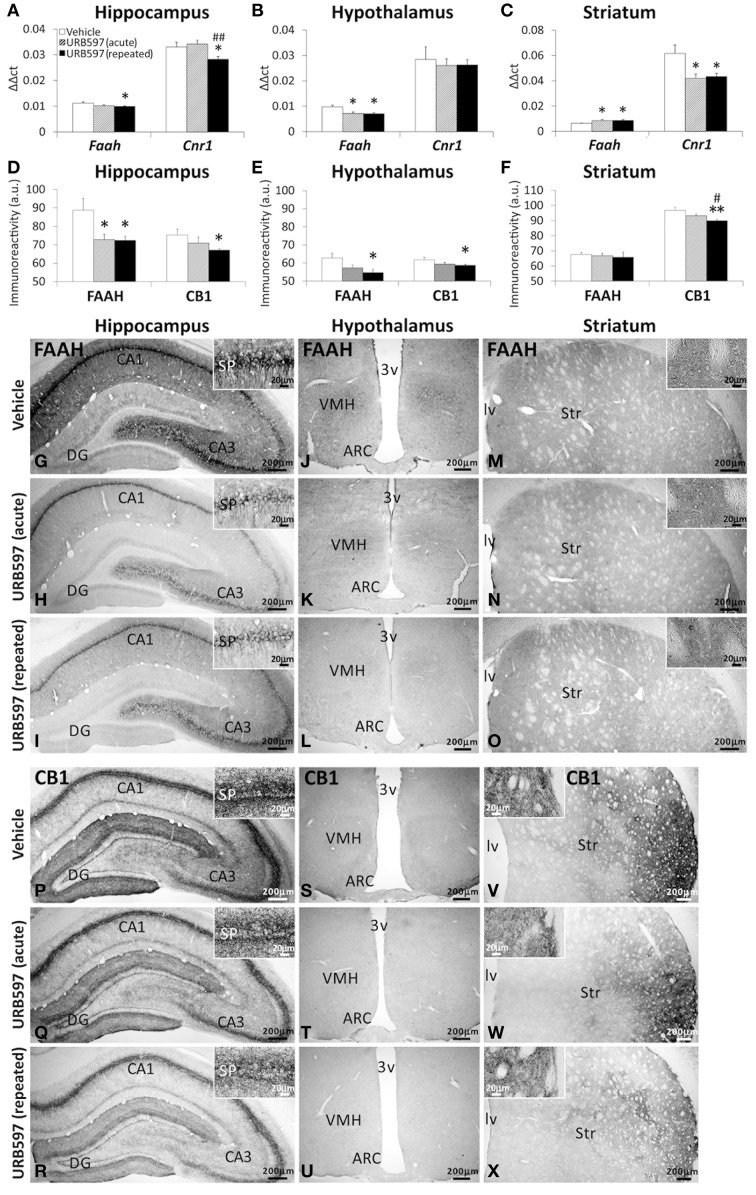
**Gene (A–C) and immunohistochemical (D–F) expression of FAAH and CB1 receptor in the hippocampus, hypothalamus and striatum of acute and repeated URB597-treated rats**. **(G–X)** Representative microphotographs showing low and high (insets) magnification views of FAAH and CB1 receptor immunoreactivity in the hippocampus, hypothalamus and striatum. The histograms represents the mean ± s.e.m. (*n* = 6–8) per experimental group. ANOVA and Bonferroni's test: ^*^P < 0.05, ^**^P < 0.01 vs. vehicle group, ^#^*P* < 0.05, ^##^*P* < 0.01 vs. acute URB597 group.

To assess whether the changes observed in the endocannabinoid tone are in concordance with the expression of pertinent components of the endocannabinoid system, we evaluated the gene and protein (immunohistochemical) expression of the NAEs-hydrolyzing enzyme FAAH and the CB1 cannabinoid receptor in the hippocampus, hypothalamus and striatum of rats after URB597 treatment (Figure 9). Rats repeatedly treated with URB597 showed decreases in both gene and immunohistochemical expressions of FAAH and CB1 in the hippocampus (Figures 9A,D,G–I,P–R), FAAH in the hypothalamus (Figures 9B,E,J–L) and CB1 in the striatum (Figures 9C,F,V–X). In addition, we observed additional changes in gene expression, which is not confirmed by the correspondent immunohistochemical analysis, such as increased levels of *Faah* mRNA in the striatum of acute and repeated URB597-treated rats as well as the decrease in the hypothalamic levels of *Faah* mRNA and the striatal levels of *Cnr1* mRNA in acute URB597-treated rats (Figures 9B,C).

## Discussion

The biological activities associated to the NAEs OEA, PEA, and AEA, including neural proliferation, cell survival and neuroinflammation, require the control of their levels, for instance through the NAEs hydrolyzing FAAH activity, and the activation of their local targets, such as the cannabinoid, TRPV1 and PPARα receptors. Our results confirm that the selective FAAH inhibitor URB597 administered at an effective dose of 0.3 mg/kg/day for 5 days increased the levels of OEA, PEA and AEA in the rat plasma. Different effects on the local expression of FAAH and CB1 receptor were observed depending on the brain region analyzed. In these rats, FAAH expression was specifically reduced in the hippocampus and hypothalamus, whereas CB1 expression was specifically reduced in the hippocampus and striatum. The experimental group of rats sacrificed 5 days after the acute treatment with URB597 (one dose and 4-days resting) was performed in order to analyze whether the effects of the acute URB597 treatment were persistent or compensatory along time. These rats showed reductions of the immunohistochemical or gene expression of FAAH in the hippocampus and hypothalamus as well as a decrease of CB1 gene expression in the striatum. In keeping to the decreased expression of FAAH obtained in the brain of URB597-treated rats, previous studies showed that the genetic deletion of *faah* gene in mice and the systemic administration of URB597 in rats elevated brain levels of AEA and amplified the responses of this endogenous cannabinoid agonist (Fegley et al., 2005; Piomelli et al., 2006). Thus, the brain of *faah*-null mice showed an increase that ranged from 2 to 15-fold of AEA levels, a 17-fold increase of OEA levels and a 8-fold increase of PEA levels (Cravatt et al., 2001; Fegley et al., 2005). One i.p. administration of URB597 at a dose of 0.3 mg/kg produced a 1.3-fold increase of AEA and 1.6-fold increase of OEA and PEA in the mouse brain (Fegley et al., 2005). Our results indicate that the repeated administration of URB597 for 5 days can produce specific alterations in the local expression of CB1 receptor that must be interpreted depending on the brain region analyzed. The excess of plasma levels of AEA produced by the prolonged FAAH inactivation, as a consequence of the repeated URB597 administration, can produce a saturating effect on the CB1 activity, resulting in a reduction of the turnover of CB1 receptor expression. This effect can be accepted in those brain regions with high expression of CB1 such as the hippocampus and striatum.

The present study indicate that the endocannabinoid system can actively regulate cell proliferation *in vivo*, as the effect of URB597 on the FAAH activity and expression were accompanied with changes in neural proliferation in a neurogenic region-dependent manner. Thus, the subgranular zone of the dentate gyrus of repeated URB597-treated rats showed a reduction in neural proliferation, as the number of phospho-H3^+^ and BrdU^+^ cells was decreased. These results in the hippocampus are not consistent with the promotion of cell proliferation after the specific CB1 activation and the inhibition of cell proliferation after CB1 and CB2 blockage observed *in vitro* and *in vivo* in the mouse dentate gyrus (Aguado et al., 2005; Goncalves et al., 2008). This discrepancy can be explained by (1) the down-expression of the hippocampal CB1 receptors, and (2) the putative participation of non-cannabinoid systems as a consequence of the activation of additional AEA targets such as TRPV1 receptor and/or the over-activation of OEA and PEA targets such as PPARα receptors. In agreement with our results, it should be mentioned that (a) TRPV1-null mice showed an improved neurogenesis in the SGZ, but not in the SVZ, after spatial learning (unpublished observations, dissertation by Stock K., 2012; see also Stock et al., 2012); and (b) URB597 administered at full inhibition of spinal FAAH may contribute at producing TRPV1-mediated analgesia (Starowicz et al., 2013). On the contrary, the subventricular zone of the lateral ventricles of acute URB597-treated rats showed an increase of the neural proliferation, as the number of phospho-H3^+^ and BrdU^+^ cells was augmented. The increased subventricular cell proliferation is consistent with previous studies showing a higher number of subventricular Ki67-positive cells in mice treated with URB597 at a dose of 5 mg/kg/day for 5 days and a lower number of subventricular Ki67-positive cells in mice treated with CB2 antagonists (Goncalves et al., 2008). Moreover, Goncalves et al. (2008) also showed that the effect of URB597 on the increased cell proliferation in the mouse subventricular zone was specifically blocked when mice were also treated with the selective CB2 antagonist JTE-907.

The effects of repeated URB597 on the hypothalamus showed a more complicated interpretation than those on the hippocampus and striatum, as there is no previous information about the role of NAEs in the hypothalamic neural proliferation. We suggest that the specific decrease of FAAH expression in the hypothalamus could be associated with a likely decrease in the hypothalamic neural proliferation, as the number of phospho-H3^+^ cells was reduced but not the number of BrdU^+^ cells in the repeated URB597-treated rats. We suspect that it could be necessary additional days of URB597 treatment and/or a prolonged stimulation of non-cannabinoid targets such as TRPV1 or PPARα receptors to observe a clear decline in the hypothalamic neural proliferation. Considering this possibility, we evaluated the survived BrdU^+^ cells in rats sacrificed 3 weeks after administering BrdU and URB597. Interestingly, we could detect a decrease in the number of BrdU^+^ cells specifically in the hypothalamus, but not the remaining brain areas analyzed, of the repeated URB597-treated rats. In contrast to these data, the hypothalamus of the rats sacrificed 5 days after the acute URB597 treatment showed a residual increase in neural proliferation, as the number of BrdU^+^ cells was augmented but not the number of phospho-H3^+^ cells. We suspect that the acute URB597 effect on the hypothalamic neural proliferation was erased as a consequence of the four resting days after the treatment. Regarding these previous results, we suggest that AEA-TRPV1 and OEA/PEA-PPARα signaling could be relevant pathways to be considered when analyzing neurogenesis in the adult brain.

Beside the likely association between the local expression of FAAH and the adult neurogenic cell proliferation, we hypothesized that processes related with neuroinflammation and neuroprotection can be primary influence by the plasma levels of the endocannabinoids (Balvers et al., 2013). Thus, the effects of URB597 on the plasma levels of AEA, OEA, and PEA were accompanied with changes in the glia and apoptosis in a brain region-dependent manner. The hippocampus of repeated URB597-treated rats showed a decrease in the number of cells expressing GFAP (astrocytes), Iba-1 (microglial cells) and cleaved caspase-3 (apoptotic cells). A similar reduction in glia and apoptosis was observed in the hippocampus of rats sacrificed 5 days after the acute URB597 treatment. Regarding the hypothalamic glia, we observed a reduction in the number of cells expressing GFAP but not Iba-1, suggesting a reduction of astrocytes but not microglial cells in the hypothalamus of rats acutely and repeatedly treated with URB597. These results indicate that URB597 treatment likely showed an anti-inflammatory profile, being evident in the hippocampus and hypothalamus, which can be attributed to the high levels of PEA in the plasma. URB597 and, specifically, PEA play an important role in neuroinflammation propagation, as they can attenuate microglia activation and migration by interacting with cannabinoid-like receptors (Muccioli and Stella, 2008; Murphy et al., 2012). On the contrary, the striatum of acute URB597-treated rats showed an increase in the number of cells expressing GFAP (astrocytes) and cleaved caspase-3 (apoptotic cells). This opposite effect in the striatum can be produced as a consequence of a compensatory effect during the resting days after the treatment.

It should be also noted a negative energy balance in rats repeatedly treated with URB597, as we observed a transitory reduction of body weight that was accompanied with a decrease in the plasma levels of the basal glucose, tryglicerides, cholesterol, leptin, and insulin. Despite the fact that no long-term modification in cumulative food intake was detected in these rats, the amelioration of these metabolic parameters agrees with the central effect of feeding-controlled signals. Thus, the increased levels of OEA can produce anorexic effects by activating satiety signals in the hypothalamus (Fu et al., 2003; Lo Verme et al., 2005a) and could be related with a reduced number of hypothalamic proliferating cells.

As summary, the repeated administration of URB597 increased the plasma levels of AEA, OEA, and PEA, and produced decreased expression of FAAH and CB1 receptor in the hippocampus, hypothalamus and striatum. This endocannabinoid tone, as a consequence of the URB597 treatment, is coupled to a decrease in neurogenic cell proliferation, cell survival and glia, mainly focalized in the hippocampus and hypothalamus, as well as a reduced apoptosis in the hippocampus and striatum. In contrast, one dose/4-days resting of URB597 treatment produced opposed effects specifically observed in the striatum. These changes in the brain were associated with a negative energy balance as a transitory body weight decrease and reduced plasma levels of glucose, triglycerides and cholesterol were observed.

## Author contributions

All authors had full access to all the data in the study and take responsibility for the integrity of the data and the accuracy of the data analysis. Study concept and design: PR, FR, JS. Acquisition of data: PR, LB, AP, M, FP, AS, RT, BL, FR, JS. Analysis and interpretation of data: PR, FR, JS Drafting of the manuscript: FR, JS. Critical revision of the manuscript for important intellectual content, obtained funding and study supervision: BL, FR, JS.

### Conflict of interest statement

The authors declare that the research was conducted in the absence of any commercial or financial relationships that could be construed as a potential conflict of interest.

## Abbreviations

3v: third ventricle
ARC: arcuate nucleus of hypothalamus
BrdU: 5-bromo-2′-deoxyuridine
CA1/3: field 1/3 of Cornu Ammonis
CB1/2: cannabinoid type 1 and 2 receptor
*Cnr1*: cannabinoid CB1 receptor gene
DG: dentate gyrus
EPl: external plexiform layer
gcl: dentate granular cell layer
GrO: granular cell layer of the olfactory bulb
FAAH: fatty acid amide hydrolase
GFAP: glial fibrillary acidic protein
HOMA-IR/β: homeostatic model assessment, insulin resistance/β-cell function
ml: molecular layer
Iba-1: ionized calcium-binding adapter molecule 1
IPl: internal plexiform layer
lv: lateral ventricle
NeuN: hexaribonucleotide binding protein-3
pcl: polymorphic cell layer
PVH: paraventricular nucleus of hypothalamus
SGZ: subgranular zone of dentate gyrus
Str: striatum
SVZ: subventricular zone of the lateral ventricles
SL-M: stratum lacunosum-moleculare
SO: stratum oriens
SP: stratum pyramidale
SR: stratum radiatum
URB597: cyclohexyl carbamic acid 3′-carbamoyl-biphenyl-3-yl ester
VMH: ventromedial nucleus of hypothalamus.
