# Supplementary material for: Pharmacological blockade of the fatty acid amide hydrolase (FAAH) alters neural proliferation, apoptosis and gliosis in the rat hippocampus, hypothalamus and striatum in a negative energy context
Source: Front Cell Neurosci. 2015 Mar 27;9:98. doi: 10.3389/fncel.2015.00098 (PMC4375993; doi:10.3389/fncel.2015.00098)
Supplement: Supplementary file 1 [file DataSheet1.PDF]

SUPPLEMENTARY MATERIALS AND RESULTS

Materials and methods

Dose-response treatment of URB597

FAAH inhibitor URB597 [(3'-(aminocarbonyl)[1,1'-biphenyl]-3-yl)-cyclohexylcarbamate; Cayman Chemical, cat. no. 10046, Ann Arbor, MI, USA] was dissolved in a vehicle containing 33% (*v*/*v*) DMSO in sterile 0.9% NaCl solution, just before each experiment. The vehicle or URB597 were injected intraperitoneally (i.p.) in a final volume of 1 ml/kg body weight. URB597 was acutely administrated at doses of 0.03, 0.3 and 3 mg/kg body weight. For the acute dose-response treatment of URB597, the cumulative food intake, body weight gain, body temperature and glucose tolerance were measured over a time course that ranges from 15 minutes to 24 hours, depending on the parameter analyzed, in rats previously food-deprived for 12 hours (n=8). The optimal dose at which acute treatment would be more effective was selected for the repeated treatment experiment.

Measurement of temperature

Body temperature was recorded using a rectal probe for rodent connected to a handheld digital thermometer (BAT-12 Microprobe-Thermometer, Physitemp). For each animal, three or four records were taken over a time course of 0, 0.5, 3, 6 and 24 hours after the acute treatment of URB597 (0.03, 0.3 and 3 mg/kg) or vehicle (n=8).

Glucose tolerance test

At 30 minutes before starting the glucose tolerance test (GTT), 0.03, 0.3 and 3 mg/kg of URB597 or vehicle were administered in 12-hour fasted rats (n=8). Thirty minutes later, tail blood samples were subsequently collected at basal level (0 minutes) and 15, 30, 45,

60 and 120 minutes after a glucose overload (i.p.) at a dose of 2 g/kg body weight. Glucose levels were determined using a standard glucose oxidase method.

Results

Acute treatment of URB597 at doses of 0.03, 0.3 and 3 mg/kg of body weight were used for the analysis of the dose-response on cumulative food intake, body weight gain, body temperature and glucose tolerance over the appropriate time course (Fig. S1). The optimal dose at which acute treatment would be more effective was selected for the repeated treatment experiment. URB597 at a dose of 0.03 mg/kg showed a reduction of cumulative food intake for 30 minutes after treatment ($P<0.01$) (Fig. S1A). URB597 at a dose of 0.3 mg/kg showed a reduction in the cumulative food intake for 30 ($P<0.01$), 60 ($P<0.01$), 120 ($P<0.05$) and 240 ($P<0.01$) minutes after treatment. URB597 at a dose of 3 mg/kg showed a reduction in the cumulative food intake for 30 ($P<0.01$), 60 ($P<0.05$) and 120 ($P<0.05$) minutes after treatment. Neither of the doses used for URB597 showed any effect on cumulative food intake for 24 hours after treatment (Fig. S1A).

Only the rats with acute treatment of URB597 at a dose of 0.3 mg/kg showed a transitory decrease in their body weight gain for 6 hours after treatment (*$P<0.05$) (Fig. S1B). Neither of the doses used for URB597 showed any effect on body weight gain for 24 hours after treatment (Fig. S1B).

URB597 at a dose of 0.3 mg/kg showed an increase in the body temperature for 30 min, 3 h and 6 h ($P<0.05$) minutes after treatment (Fig. S1C). URB597 at doses of 0.03 and 3 mg/kg showed an increase of body temperature for 6 hours after treatment ($P<0.05$) (Fig. S1C).

No change in glucose tolerance was observed in rats treated with URB597 at doses of 0.03 and 0.3 mg/kg (Fig. S1D). However, URB597 treatment at a dose of 3 mg/kg showed an increase in plasma levels of glucose detected 45 minutes after the glucose overload (Fig. S1D).

Figure S1. Dose-response of acute treatment of URB595 (0.03, 0.3 and 3 mg/kg) on relative food intake (**A**), body weight gain (**B**), body temperature (**C**) and glucose tolerance (**D**). The histograms represents the mean ± s.e.m. (*n*=8) per experimental group. ANOVA and Bonferroni's test: **P*<0.05, ***P*<0.01 *vs*. vehicle group.

Figure S2. Time line of the experimental design that summarize the treatments and experimental groups used in the present study.
