# Supplementary figures and images for: Pharmacological blockade of the fatty acid amide hydrolase (FAAH) alters neural proliferation, apoptosis and gliosis in the rat hippocampus, hypothalamus and striatum in a negative energy context
Source: Front Cell Neurosci. 2015 Mar 27;9:98. doi: 10.3389/fncel.2015.00098 (PMC4375993; doi:10.3389/fncel.2015.00098)

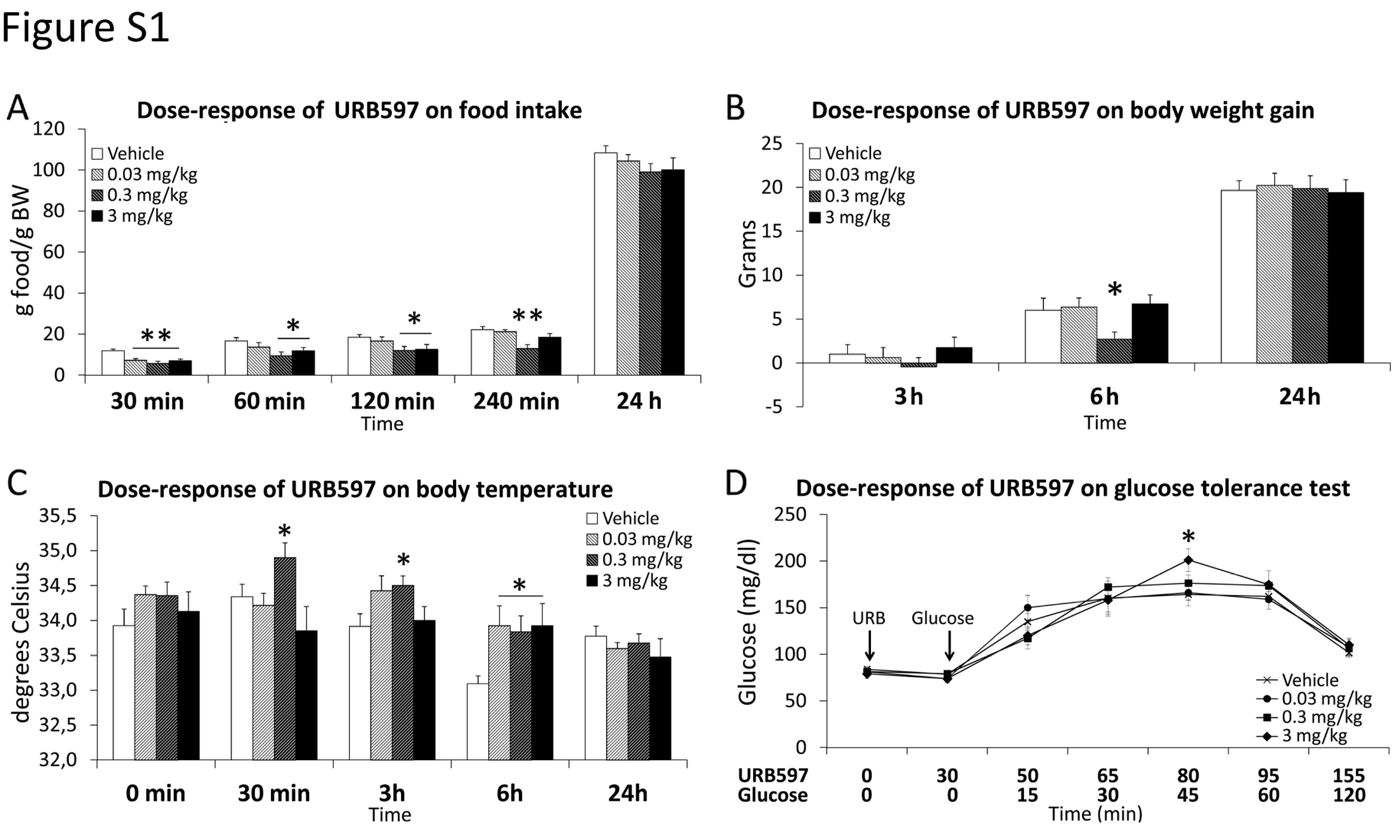

Supplement: Supplementary file 2 [file Image1.TIF]

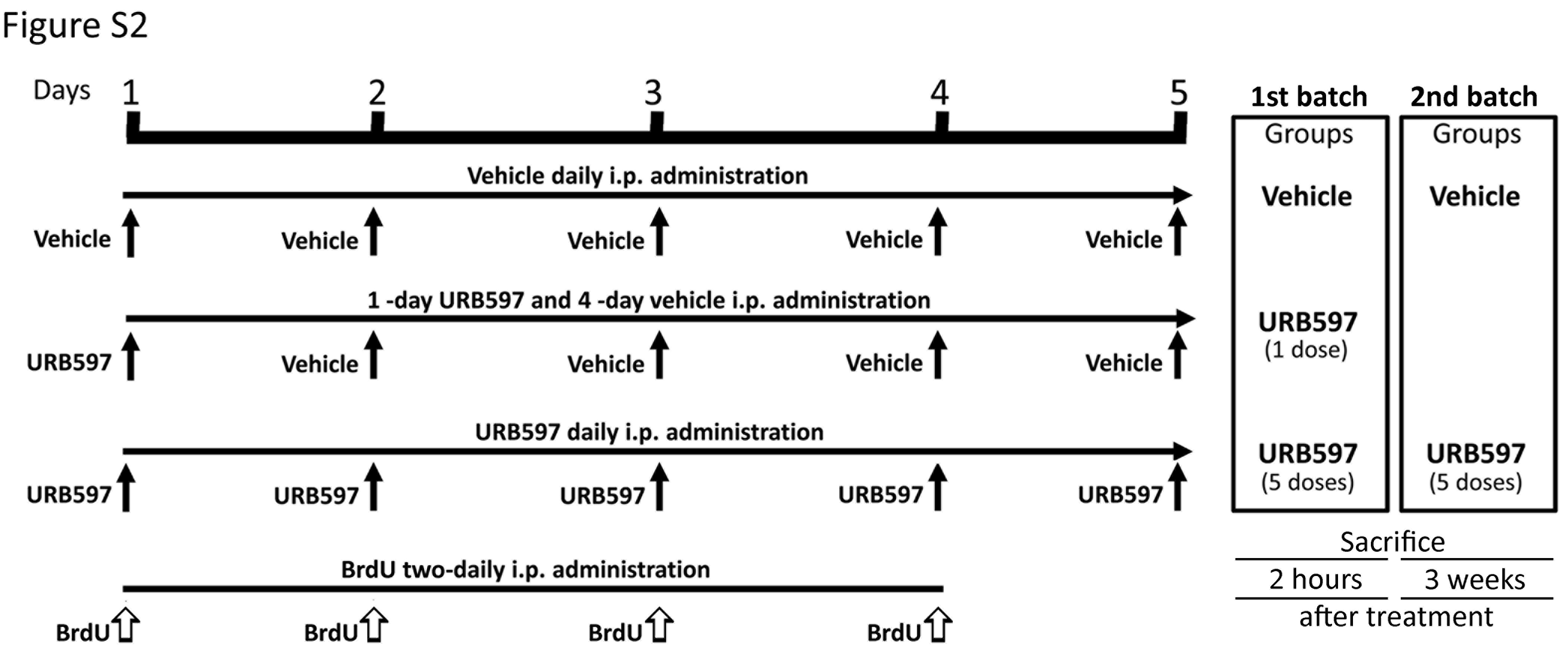

Supplement: Supplementary file 3 [file Image2.TIF]
